# Functional Outcome of Extensor Mechanism Salvage in Patella Fracture Non-union Using Krackow Sutures: A Case Report

**DOI:** 10.7759/cureus.73808

**Published:** 2024-11-16

**Authors:** Nitin S Patil, Ami R Maru

**Affiliations:** 1 Department of Orthopedics, Krishna Vishwa Vidyapeeth (Deemed to be University), Satara, IND

**Keywords:** extensor mechanism, krackow suturing, patellar non-union, salvage procedure, tension band wiring

## Abstract

Patellar fractures can lead to extensor mechanism dysfunction if not repaired properly, impacting knee function and mobility, and this complication can be challenging to manage, especially in cases where previous surgical interventions have failed. The aim of this study was to evaluate the functional outcome of the salvaged extensor mechanism in patella fracture non-union using Krakow sutures in a 61-year-old female patient who presented with knee pain, reduced flexion, and a 40-degree extension lag after two previous patellar fracture surgeries using tension band wiring resulted in non-union. Despite rehabilitation attempts, her knee function remained compromised. Krakow suturing was performed to reinforce the patellar and quadriceps tendons, ensuring a secure extensor mechanism. This procedure provided a strong and reliable repair, even in compromised tissue. Post-surgery, successful salvage of the extensor mechanism was achieved, with improved knee function and mobility, significant improvement in Lysholm scores and Tegner activity levels, and 80% restoration of quadriceps strength compared to the contralateral side. This case demonstrates that Krakow suturing can be an effective salvage procedure for extensor mechanism dysfunction in patellar non-union, even in previously operated cases, restoring knee function and mobility.

## Introduction

Patellar fractures are a prevalent injury around the knee joint, accounting for approximately 1% of all skeletal fractures [1]. When the quadriceps muscle attaches to the proximal fractured fragment, it pulls proximally, creating a gap at the fracture site. A large gap can prevent fibrous union, leading to quadriceps mechanism failure and extension lag. While patients with low functional demands may tolerate non-union of the patella, surgical intervention is often necessary for active individuals [2]. The primary surgical goal is to restore the quadriceps mechanism, enabling knee extension without significantly compromising knee mobility.

Biomechanics of the extensor mechanism of the knee joint

The knee's extensor mechanism relies on a complex interplay of static and dynamic stabilizers converging on the patella. Within this framework, the patella serves as a lever arm for knee extension, effectively enhancing quadriceps force and contributing to the knee's extensor mechanism [3]. The extensor mechanism plays a vital role in maintaining an upright posture and facilitating unassisted gait. The patella's primary functions are linking and displacement [4]. During flexion, it articulates with the femoral trochlea, acting as a link between the quadriceps muscle and proximal tibia. Notably, the proximal patella's cartilage-covered surface withstands peak pressure between 45° and 60° of flexion [5]. The quadriceps tendon, comprising four muscle-tendon units, inserts on the superior pole of the patella, facilitating efficient transmission of forces.

The knee's biomechanical function relies on intricate interactions between osseous, ligamentous, and muscular structures. This complex interplay enables the knee to achieve a delicate balance between stability and mobility, with the quadriceps muscle group playing a pivotal role as both a knee extensor and stabilizer. Biomechanical studies demonstrate that the patella increases the quadriceps' effective lever arm by up to 30% at full extension, through displacement of the quadriceps-tibia linkage away from the knee rotation axis [4]. Furthermore, the patella resists knee flexion by converting tensile forces into compression forces, particularly during downhill walking or stair descent [6-8]. This phenomenon is known as the "patella-femoral joint reaction (PFJR)."

## Case presentation

**Figure 1:**
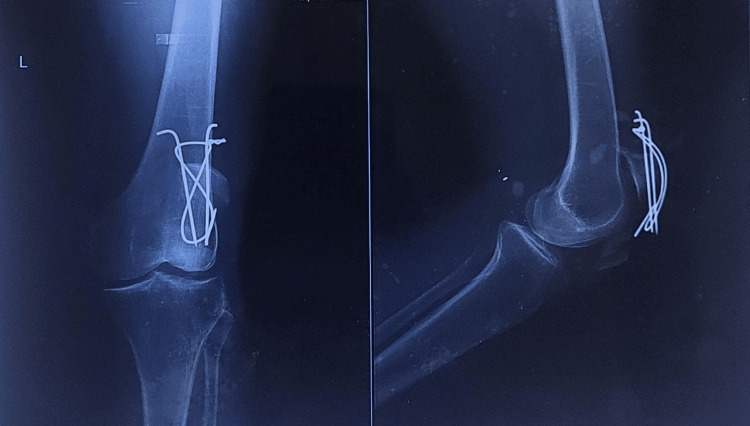
Plain radiograph obtained after first tension band wiring surgery of fractured patella. Post-operative anteroposterior and lateral knee radiographs showing patella fracture reduction and internal fixation with tension band wiring. Note the distracted fragments indicating loss of fracture reduction

**Figure 2:**
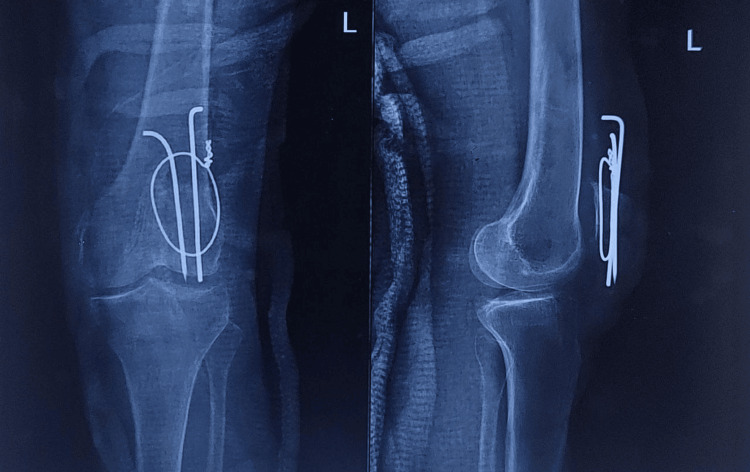
Post-operative radiograph following revision tension band wiring surgery. Post-operative anteroposterior and lateral knee radiographs illustrating improved fracture reduction, alignment, and fixation with revised tension band wiring (two weeks post-trauma)

**Figure 3:**
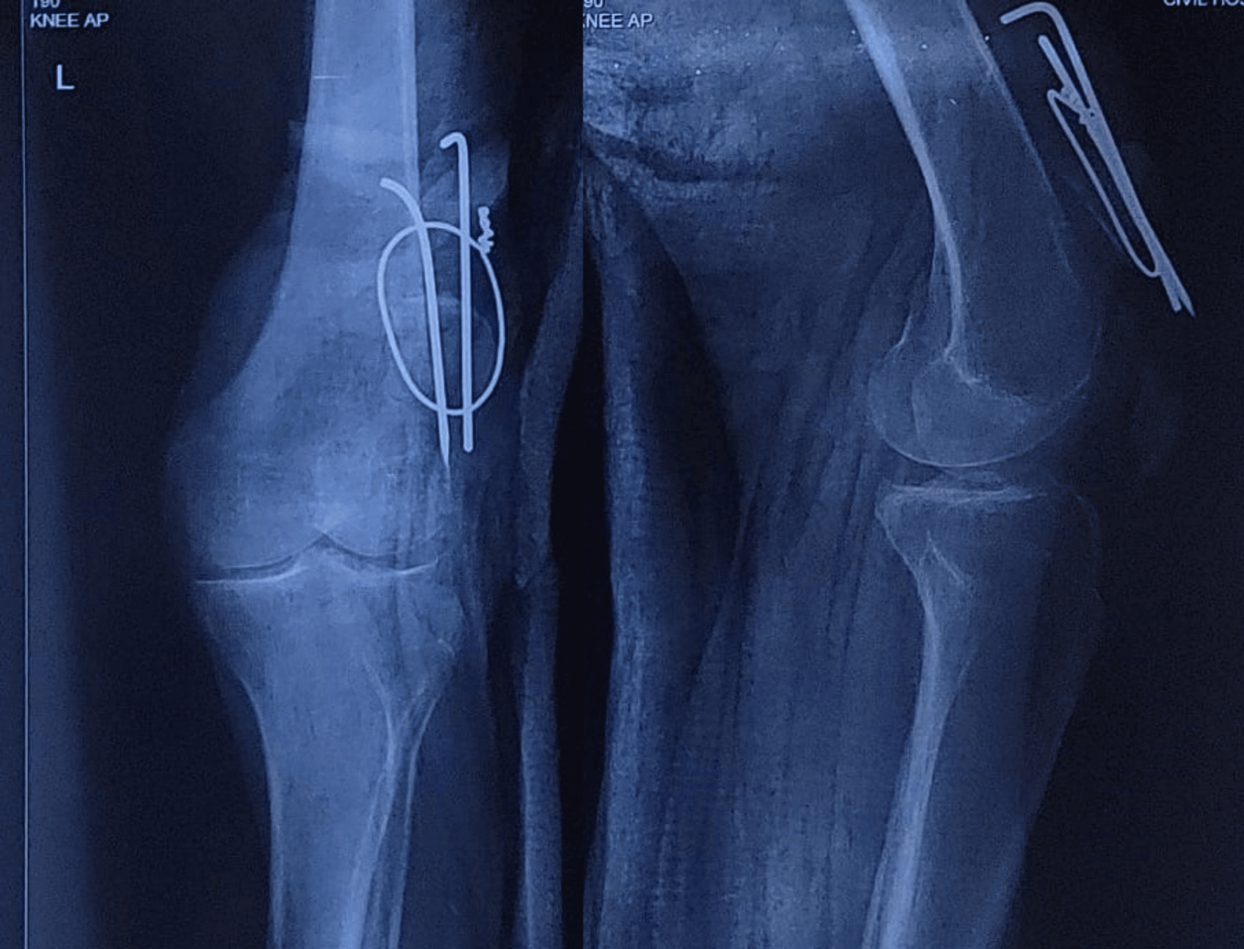
One-month post-operative follow-up radiograph. One-month post-operative anteroposterior and lateral radiographs of the knee demonstrating recurrent loss of fracture reduction and non-union of patella

**Figure 4:**
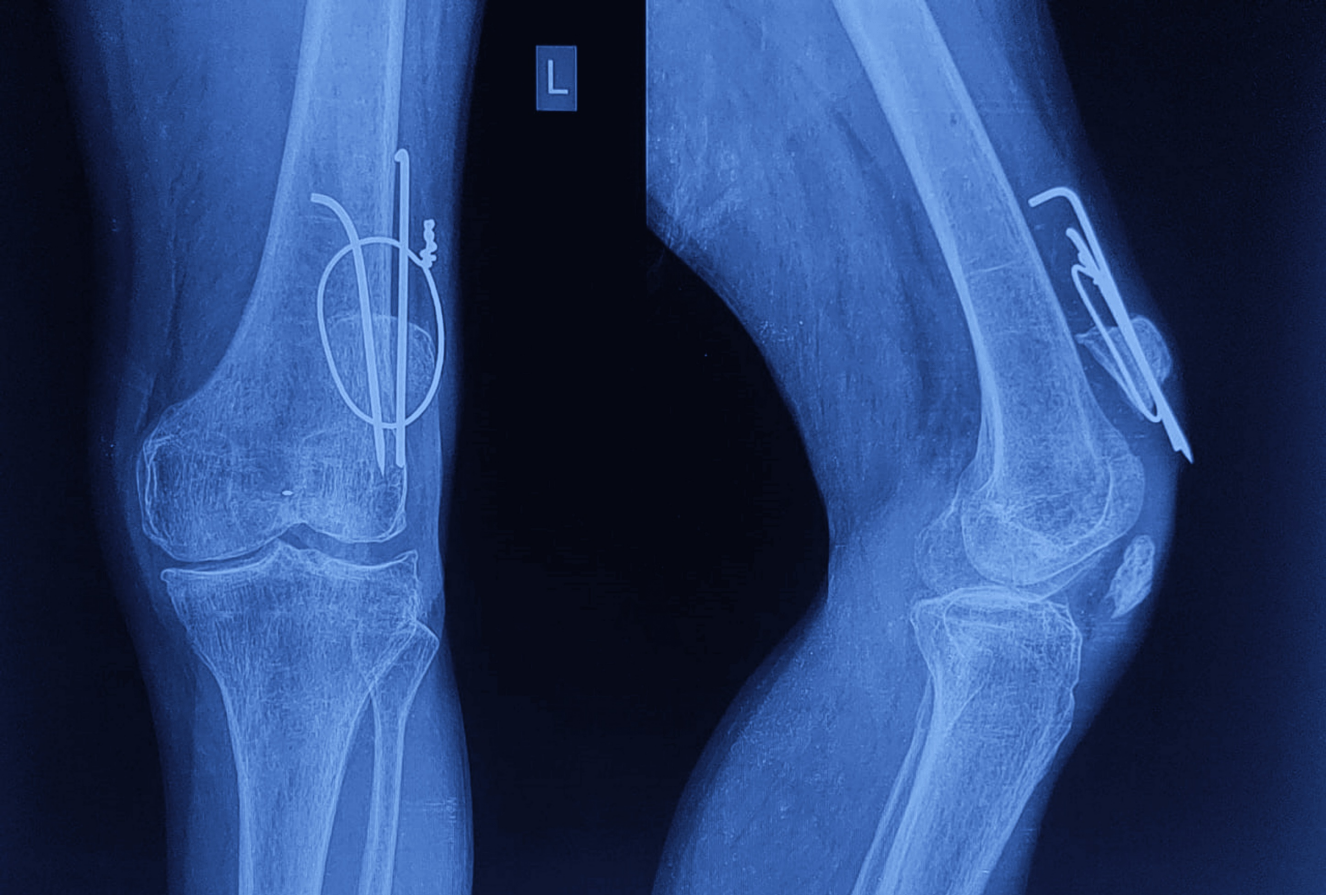
Radiographic image of the patient's left knee joint on admission. 1.5 months post-operative anteroposterior and lateral radiographs of the knee on admission at Krishna Hospital showing persistent displaced patella fragments and non-union

**Figure 5:**
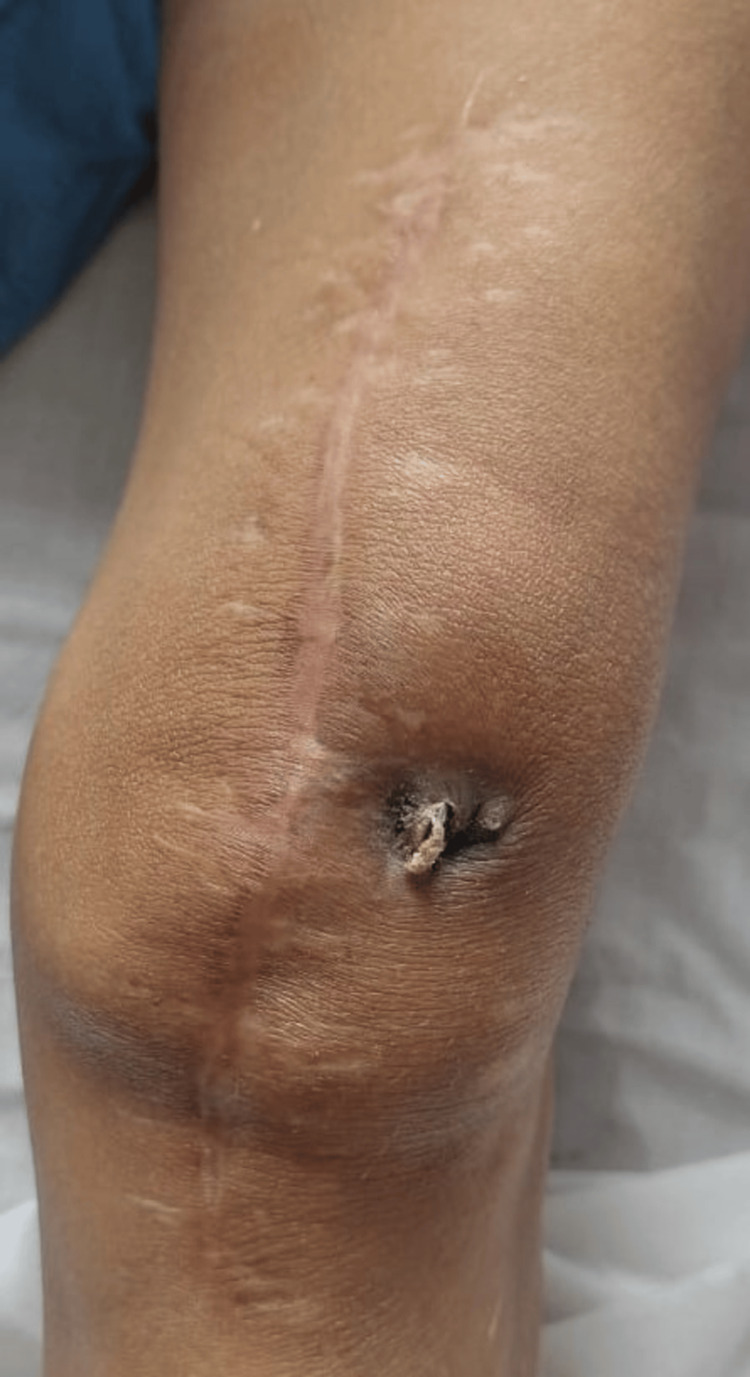
Clinical photograph of the patient's left knee at admission. Image demonstrating surgical scar of previous surgery, discharging sinus, and protruding K-wire on presentation

A 61-year-old female patient presented to the Orthopedics outpatient department with a four-month history of left knee pain, accompanied by limited flexion and inability to extend the left knee joint. Her medical history revealed a patellar fracture treated with tension band wiring two months prior, which had failed, necessitating immediate revision surgery using the same technique. Upon examination and review of radiological findings at Krishna Hospital, we diagnosed a non-union of the patellar fracture, resulting in persistent pain, restricted knee mobility, and reduced flexion with a 40° extension lag, significantly impacting the patient's daily activities, such as walking, climbing stairs, and performing routine tasks. Additionally, the patient presented with a discharging sinus and a protruding subcutaneous K-wire over the left knee, causing significant discomfort on flexion and extension of the knee joint. The patient was seeking further treatment options to address the non-union and improve her quality of life. Therefore, we admitted the patient to the ward for further evaluation and to develop a comprehensive plan for the definitive management of the non-union and associated complications. Figures 1-4 show the X-rays produced by the patient at the time of presentation, and the local skin condition is shown in Figure 5.

**Figure 6:**
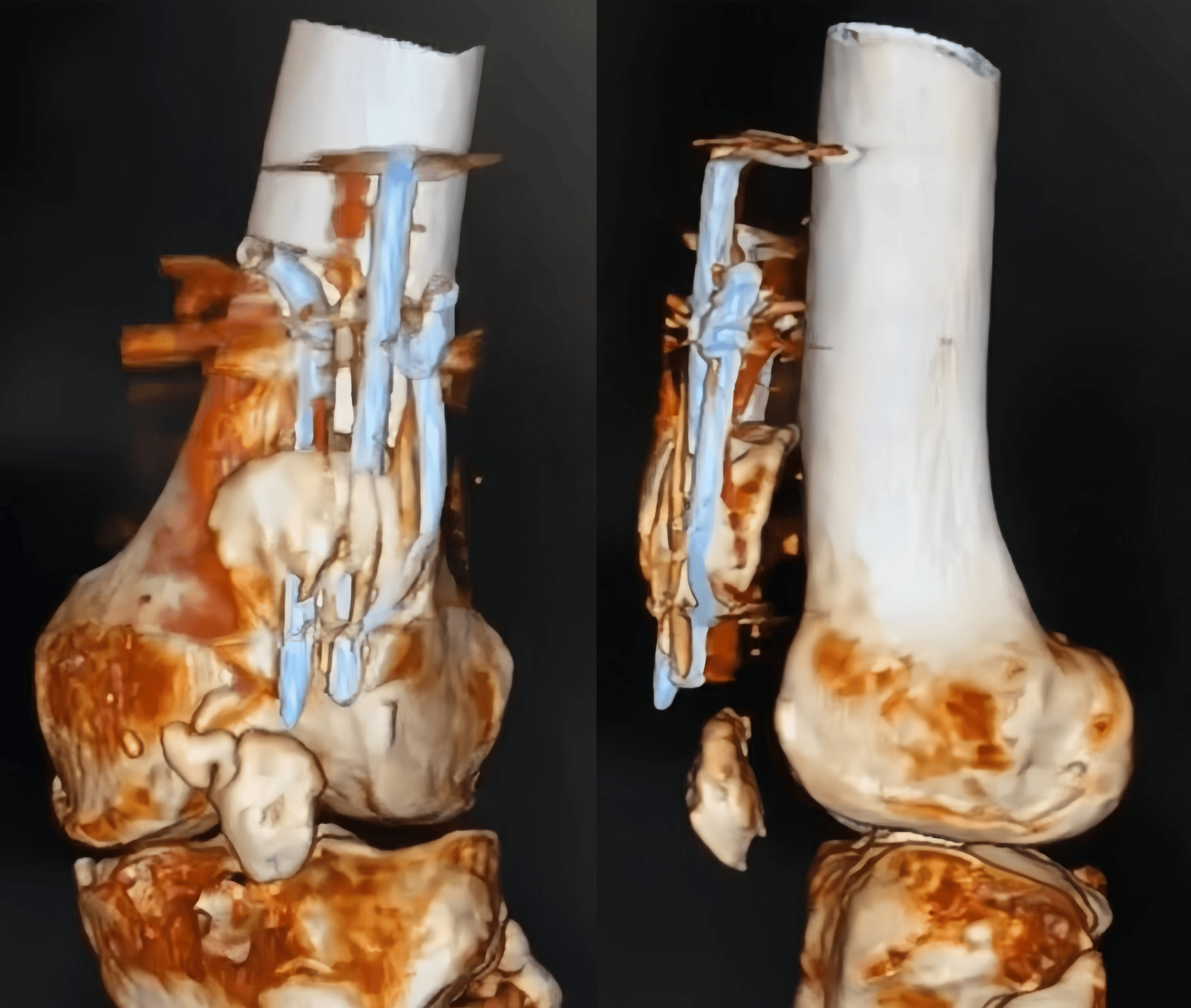
Computed tomography (CT) of the left knee joint. CT scan images showing comminuted and displaced fracture of patella leading to separation of upper and lower fragments of the patella with evident tension band wiring and K-wire fixation in the upper fragment of the patella

A computed tomography (CT) scan of the patient's left knee joint was performed, and the 3D scans were obtained as shown in Figure 6.

After admission, the patient's pre-operative preparation included a basic blood workup, medical evaluation, and anesthesia clearance. With all necessary clearances obtained, the patient was then scheduled for surgery.

Surgical procedure of extensor mechanism reconstruction with Krackow sutures

The patient underwent spinal anesthesia in the sitting position and was then placed in the supine position with the affected lower extremity in full knee extension. A tourniquet was applied, and the left lower limb was prepared for surgery through sterile scrubbing, painting, and draping. Exsanguination was achieved using an Esmarch bandage, and the tourniquet was inflated to 280 mmHg since the patient’s blood pressure was 130/80 mmHg.

**Figure 7:**
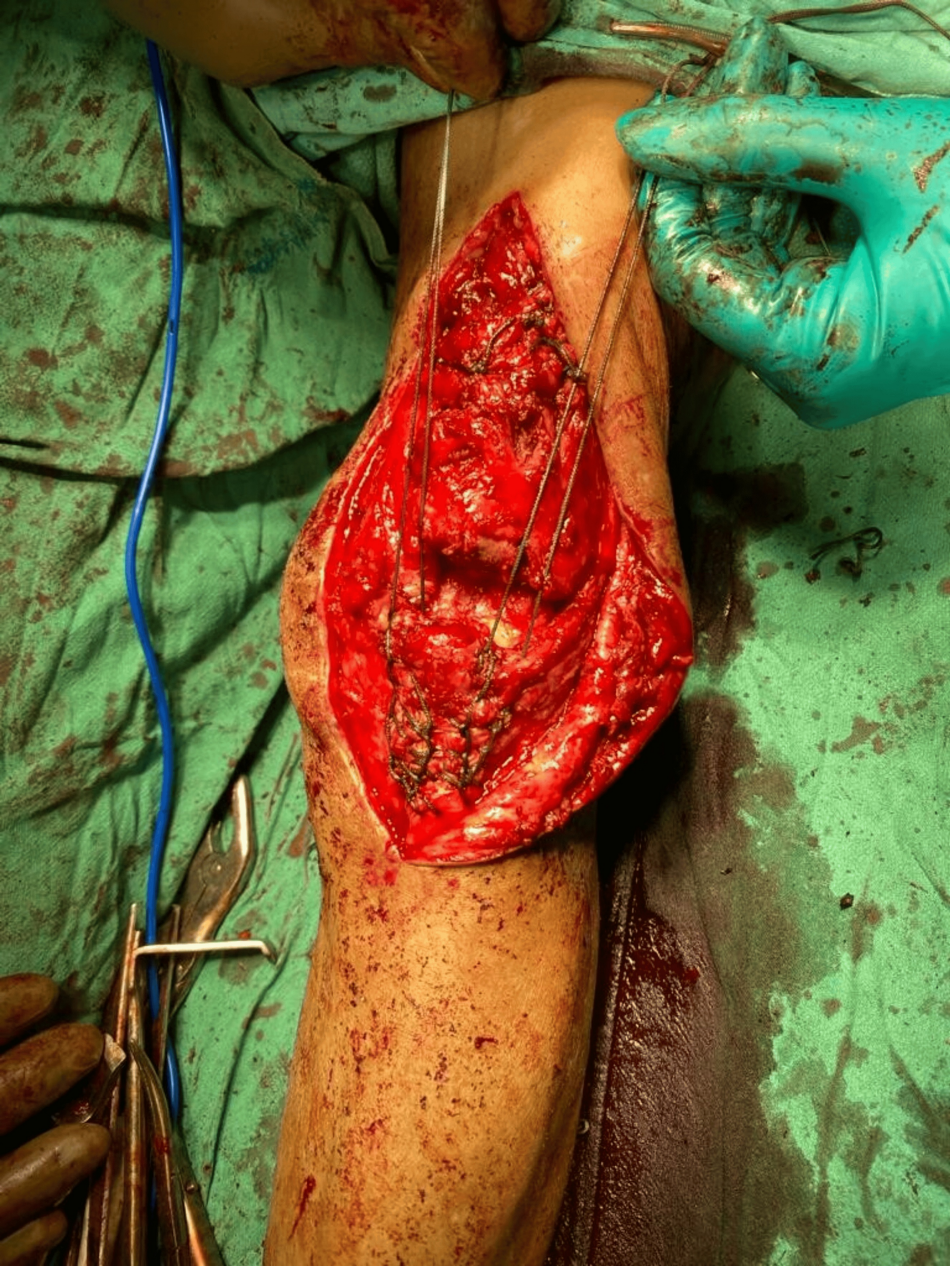
Intra-operative image of the Krackow whip stitch technique applied to both sides of the patellar tendon using #5 non-absorbable suture

A skin incision was made over the previous surgical scar, followed by deep dissection to expose the non-union of the patella. To facilitate reduction, avulsed bony fragments attached to the quadriceps or patellar tendon were utilized. The Krackow whip stitch technique was employed, involving two locking loops inserted into each side of the patellar or quadriceps tendon using the #5 non-absorbable suture as shown in Figure 7.

**Figure 8:**
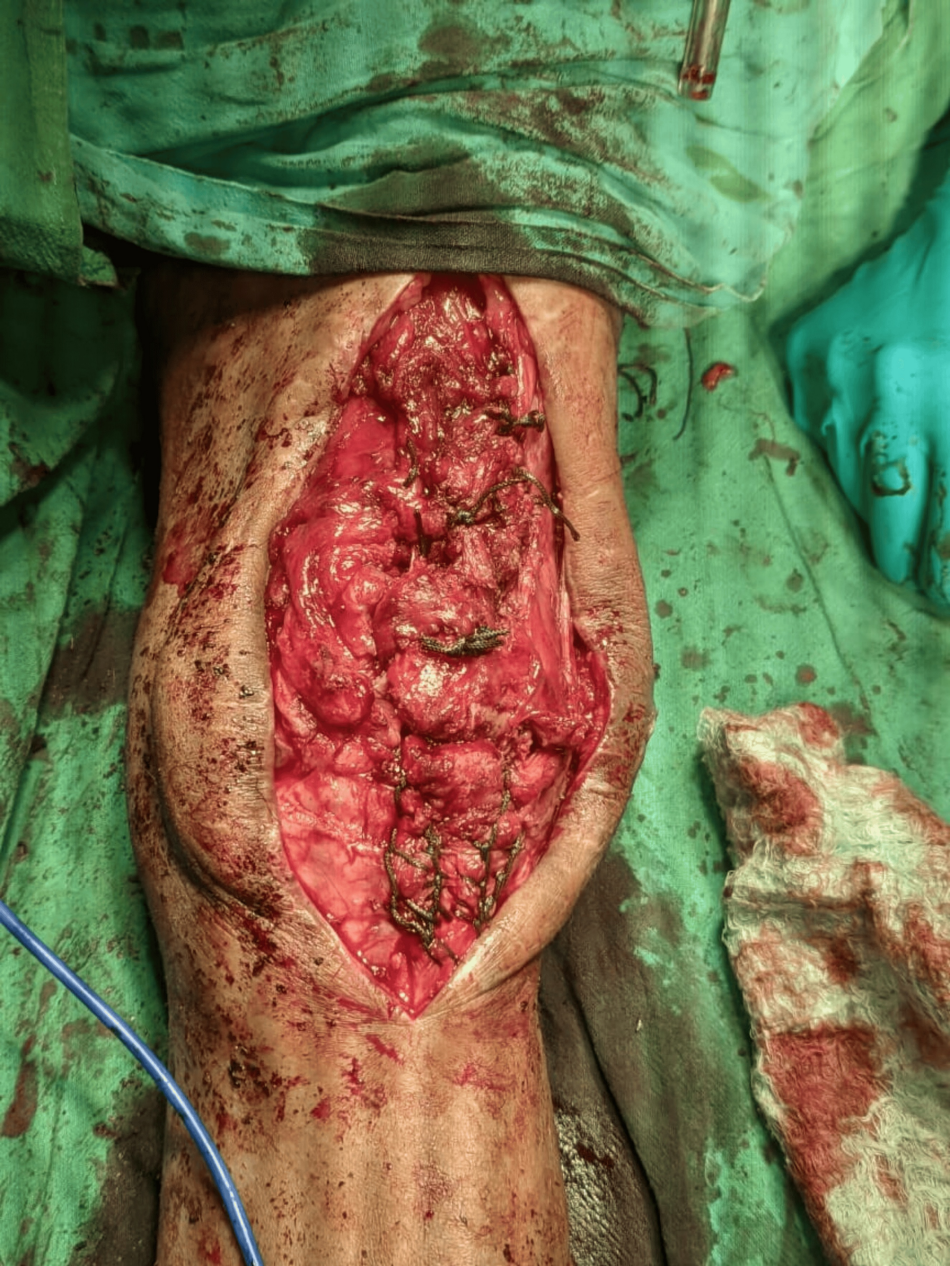
Intra-operative image of the final stage after tightening of the sutures

Patellar fragment preparation involved creating three longitudinal drill holes in the proximal patellar fragment using a 2.0-mm K-wire. Additionally, the inferior and superior poles of the patella were carefully prepared with a curette to establish a bleeding bony base, thereby maximizing the potential for healing. For ligament fixation, a suture passer was utilized to thread the free ends of the sutures through the proximal segment. The sutures were then tightened and securely tied deep to the opposing tendon, with the knee maintained in full extension, as illustrated in Figure 8.

**Figure 9:**
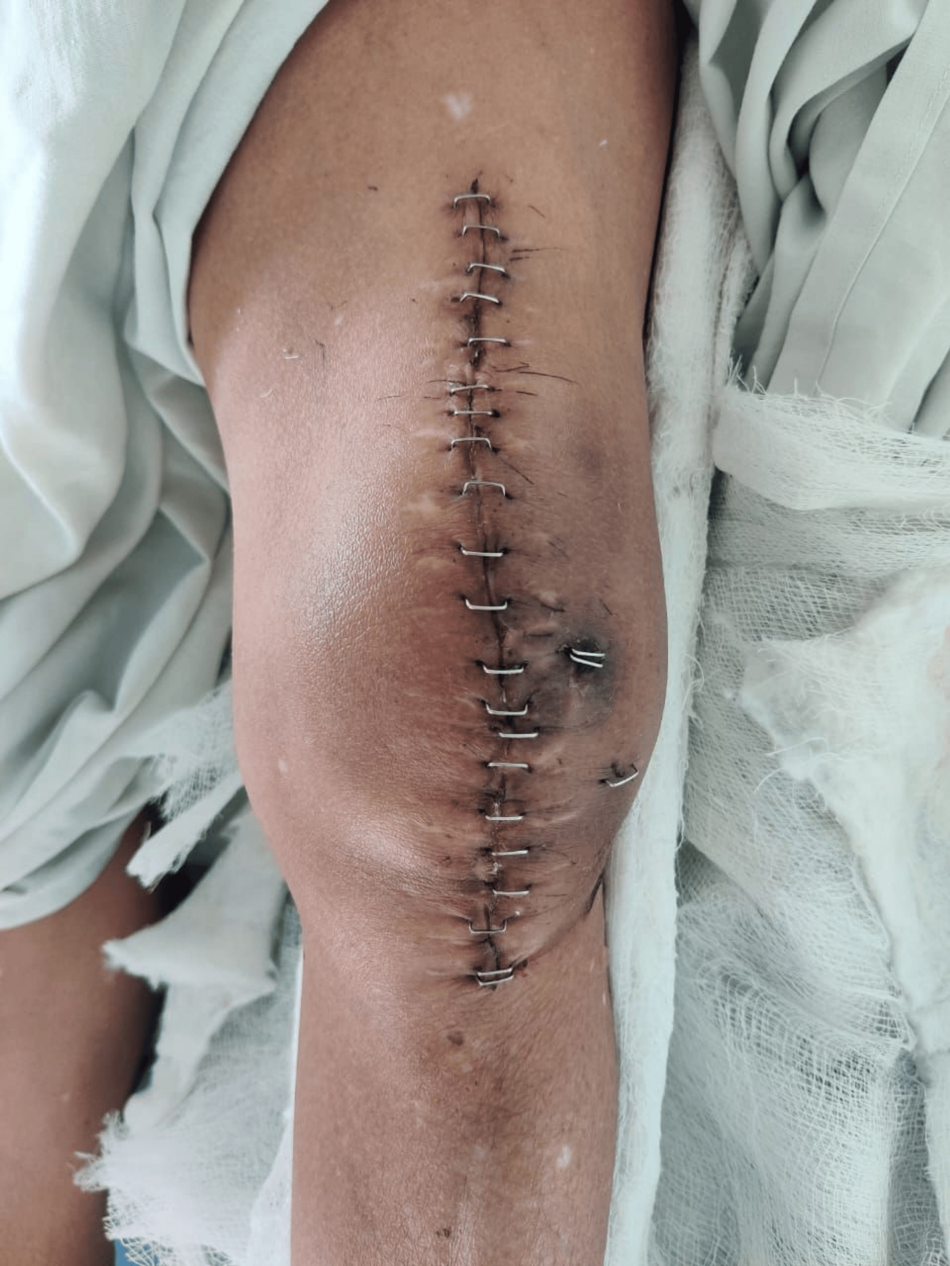
Post-operative image of the suture line

After completing the surgical procedure, the final reduction was confirmed under an image intensifier in both anteroposterior and lateral views to ensure accurate alignment. The surgical site was then thoroughly irrigated to remove any debris. Subsequent closure involved approximating the subcutaneous tissue with Vicryl 1 (Ethicon, Inc., Somerville, US) sutures, followed by skin closure using Ethilon 2-0 (Ethicon, Inc., Somerville, US) sutures, as depicted in Figure 9. This meticulous closure ensured a secure and stable environment for optimal healing.

**Figure 10:**
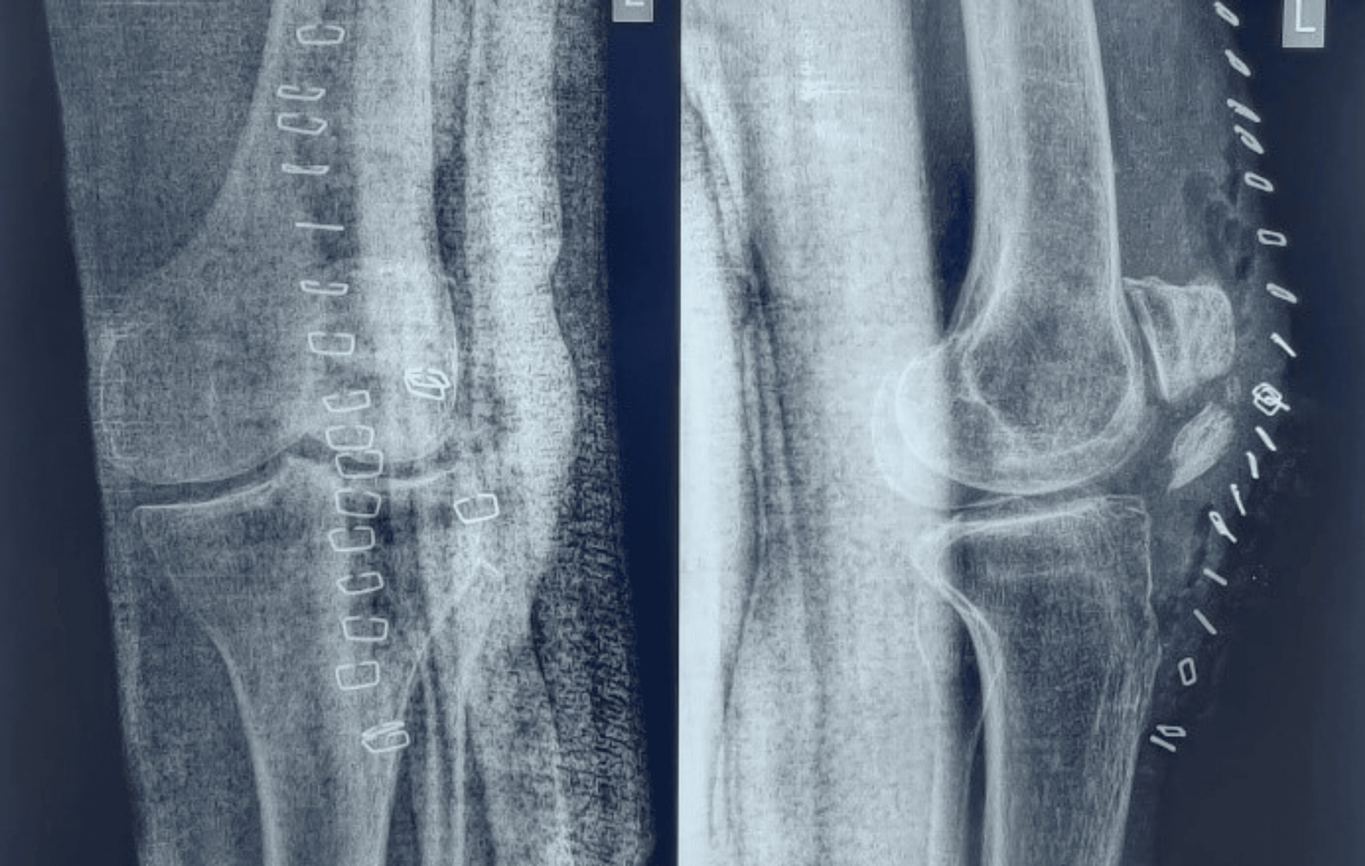
Immediate post-operative radiograph of the left knee joint after suturing the extensor mechanism by the Krackow technique

Wound dressing was done under sterile measures, and the limb was immobilized with an above-knee cylindrical slab. A post-operative radiographic image was obtained as shown in Figure 10.

**Figure 11:**
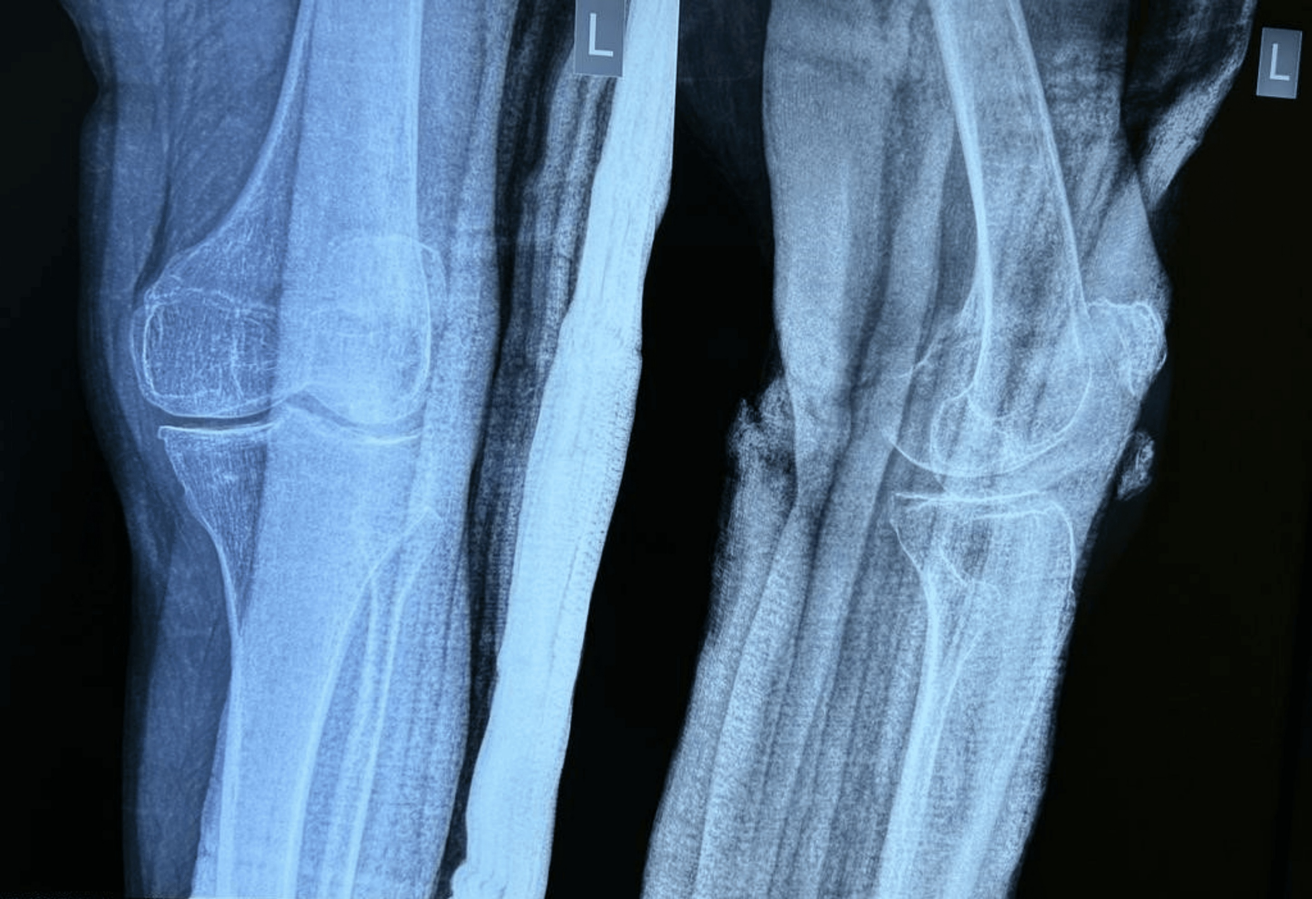
1.5-month follow-up X-ray of the left knee joint

The patient received regular follow-up care, including dressing and suture removal. At 1.5 months post-operation, a repeat X-ray was performed as shown in Figure 11, and knee range-of-motion exercises were initiated. Subsequent follow-up appointments were scheduled at regular intervals.

**Figure 12:**
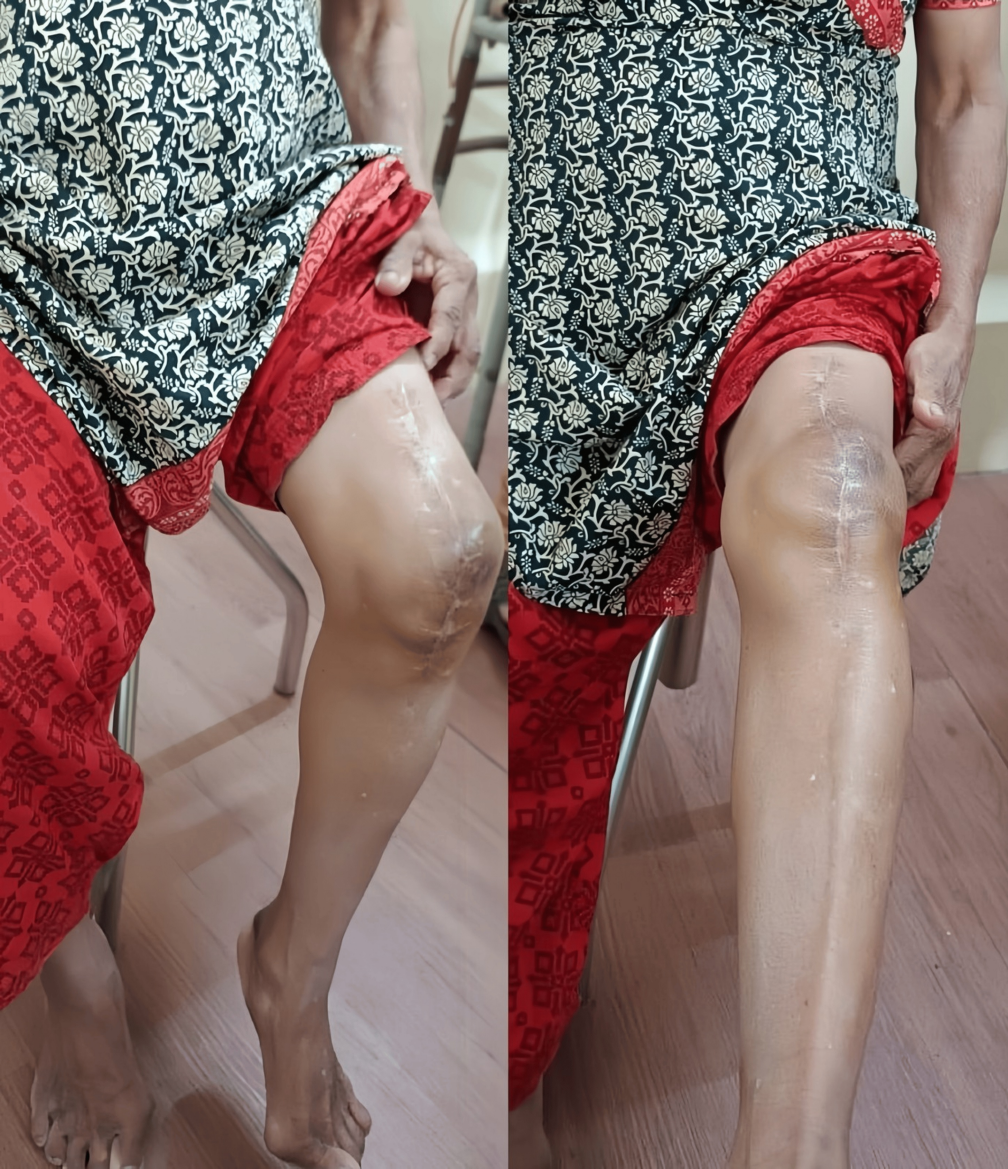
Knee flexion (70°) and extension (20°) at 1.5 months post-surgery

**Figure 13:**
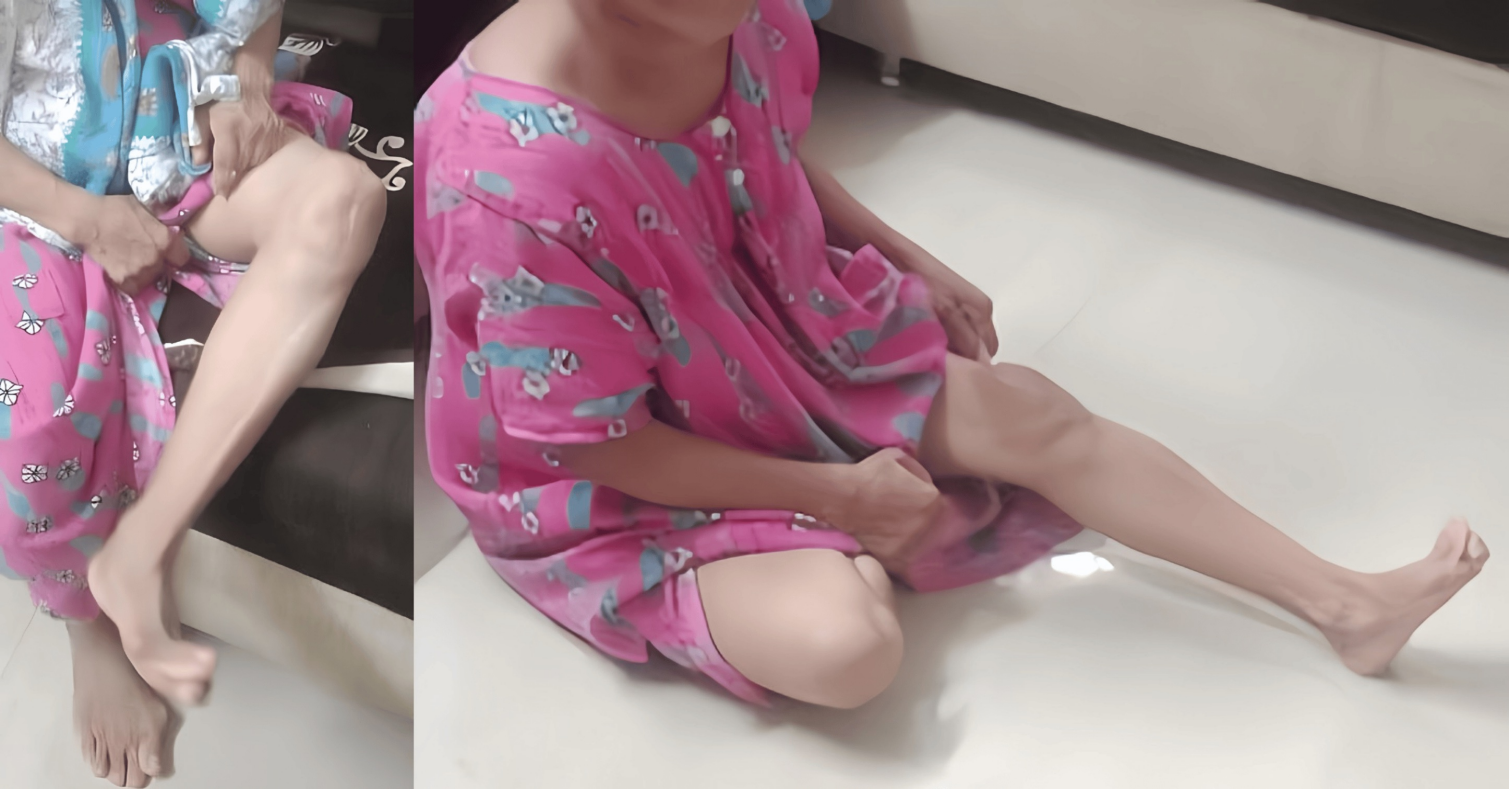
Knee flexion (100°) and full extension at six months post-surgery showing improved range of motion after extensor mechanism

In the management of patella fractures, preservation of the extensor mechanism is crucial for optimal outcomes. The successful use of Krakow sutures to salvage the extensor mechanism in a patient with a non-union of the patella highlights the importance of this approach. By maintaining the integrity of the extensor mechanism, patients can achieve significant improvements in knee function. Notably, this patient demonstrated an improved range of motion from 70° of flexion to almost 100° and extension lag reduced from 40° to almost 10°, between 1.5 and six months post-operatively as shown in Figures 12, 13.

Knee score systems, which assess factors like pain, stability, and range of motion, are essential in evaluating treatment efficacy. This case demonstrates the critical role of extensor mechanism preservation in restoring knee function and promoting recovery in patella fracture patients, as reflected in improved knee scores, enhanced range of movements, strengthened quadriceps, and increased activity levels.

## Discussion

The incidence of non-union or delayed union of patella fractures is rare, occurring in 2.7%-12.5% of cases [9]. Treatment decisions consider factors such as patient functional demands, non-union causes, potential biomechanical effects of total patellectomy, and the presence of an intact knee extensor mechanism for future reconstruction.

In tendon repair, the Krackow technique, a continuous locking loop suture method, has shown efficacy [10]. Research by Harrell et al. compared the properties of 18-gauge stainless steel wire, Mersilene (Ethicon, Inc., Somerville, US), and Ethibond (Ethicon, Inc., Somerville, US) sutures with multiple loops [11]. Their findings indicated that multiple No. 5 Ethibond sutures yielded strength similar to 18-gauge stainless steel wire. This supports the use of multiple sutures, providing better tension and stability in tendon repair, potentially reducing anterior knee pain [12].

This case study illustrates the importance of preserving the extensor mechanism in patella fractures. Employing Krakow sutures salvaged the compromised mechanism, significantly improving knee function, quadriceps strength, and range of motion. Substantial increases in the Lysholm knee score and Tegner activity level score were observed. Preserving the extensor mechanism facilitates early mobilization, reduces fracture displacement risk, and promotes uneventful healing, aligning with existing literature [9,10]. This approach highlights the critical role of extensor mechanism integrity in achieving favorable outcomes.

## Conclusions

In conclusion, this case demonstrates the effectiveness of using Krakow sutures to salvage the extensor mechanism in a patient with non-union of the patella, resulting in significantly improved knee function, range of motion, and strength. The preservation of the extensor mechanism is crucial for optimal outcomes in patella fracture management, and this technique offers a promising approach to achieving these goals. With proper surgical technique and post-operative care, patients with patella fractures, in spite of non-union of the patella, can achieve substantial improvements in knee function and quality of life, highlighting the importance of this approach in orthopedic practice.
